# A QuESt for speed: rapid qualitative evidence syntheses as a response to the COVID-19 pandemic

**DOI:** 10.1186/s13643-020-01512-5

**Published:** 2020-11-04

**Authors:** Linda Biesty, Pauline Meskell, Claire Glenton, Hannah Delaney, Mike Smalle, Andrew Booth, Xin Hui S. Chan, Declan Devane, Catherine Houghton

**Affiliations:** 1 Evidence Synthesis Ireland, National University of Ireland Galway, Galway, Ireland; 2 Qualitative Research in Trials Centre, National University of Ireland Galway, Galway, Ireland; 3 School of Nursing and Midwifery, Aras Moyola, National University of Ireland Galway, 26 Upper Newcastle, Galway, H91 E3YV Ireland; 4 Health Research Institute, University of Limerick, Limerick, Ireland; 5 Department of Nursing and Midwifery, University of Limerick, Limerick, Ireland; 6 Norwegian Institute of Public Health, Oslo, Norway; 7 School of Nursing and Midwifery, Trinity College Dublin, Dublin, Ireland; 8 James Hardiman Library, National University of Ireland Galway, Galway, Ireland; 9 School of Health and Related Research, ScHARR, University of Sheffield, Sheffield, UK; 10 Centre for Tropical Medicine and Global Health, Nuffield Department of Medicine, University of Oxford, Oxford, UK; 11 Hospital for Tropical Diseases, London, UK; 12 Cochrane Ireland, National University of Ireland Galway, Galway, Ireland; 13 HRB-Trials Methodology Research Network, National University of Ireland Galway, Galway, Ireland

**Keywords:** Rapid reviews, Qualitative evidence synthesis, Review methods, Systematic review, COVID-19

## Abstract

**Background:**

The COVID-19 pandemic has created a sense of urgency in the research community in their bid to contribute to the evidence required for healthcare policy decisions. With such urgency, researchers experience methodological challenges to maintain the rigour and transparency of their work. With this in mind, we offer reflections on our recent experience of undertaking a rapid Cochrane qualitative evidence synthesis (QES).

**Methods:**

This process paper, using a reflexive approach, describes a rapid QES prepared during, and in response to, the COVID-19 pandemic.

**Findings:**

This paper reports the methodological decisions we made and the process we undertook. We place our decisions in the context of guidance offered in relation to rapid reviews and previously conducted QESs. We highlight some of the challenges we encountered in finding the balance between the time needed for thoughtfulness and comprehensiveness whilst providing a rapid response to an urgent request for evidence.

**Conclusion:**

The need for more guidance on rapid QES remains, but such guidance needs to be based on actual worked examples and case studies. This paper and the reflections offered may provide a useful framework for others to use and further develop.

## Background

This paper describes a rapid review prepared during, and in response to, the COVID-19 pandemic. The sense of urgency that this pandemic has created in the research community has been highlighted [1], as researchers are eager to contribute to the evidence base for clinical and policy decisions. But urgency brings challenges to conducting research that maintains rigour and transparency [2]. With this in mind, we offer reflections on our experience of undertaking a rapid Cochrane qualitative evidence synthesis (QES)—‘Barriers and facilitators to healthcare workers’ adherence with infection prevention and control (IPC) guidelines for respiratory infectious diseases: a rapid qualitative evidence synthesis (QES)’ [3]. Our aim is not to offer predetermined methodological guidance, but to share with the review community the processes we used and decisions we made, so as to advance the discourse surrounding rapid QES.

In March 2020, Cochrane responded to the pandemic by beginning a process of identifying, refining and prioritising research questions to progress as Cochrane rapid reviews (see https://covidrapidreviews.cochrane.org/prioritization). Given the urgent need for evidence to inform healthcare practice and policy, Cochrane requested that COVID-19 related rapid reviews be fast-tracked and ideally completed within 2 weeks of initiation. Our review [3], featured amongst the initial suite of Cochrane COVID-19 rapid reviews, was Cochrane’s first rapid QES.

The contribution rapid reviews can make to strengthen health policy has been well-documented [4, 5]. Rapid reviews can potentially support health policy decisions by providing evidence to inform these decisions [6]. A rapid synthesis of evidence employs methods, which are ‘accelerated and streamlined’ [7] so that completion is earlier than with more typical systematic review methods [4]. As needs, not methods, determine rapid reviews, there can be no consensus on the ideal time to complete. However, from 8 weeks [8] to 12 weeks is considered usual [9]. It has been suggested that rapid models of evidence synthesis are placed ideally to ensure prompt availability of information to inform healthcare responses to emergencies [10].

The methodological guidance underpinning rapid reviews continues to advance but not without challenges [4, 11, 12]. For rapid reviews of intervention effectiveness, disagreement centres on which elements should be prioritised and which should be accelerated [5, 12]. For review authors undertaking a rapid QES, this may be even more challenging, we are only aware of one guide [13]. A scoping review, published in 2019, found an increasing number of published rapid QES (15,—13 published reviews and 2 protocols) [2]. However, the authors of this review called on rapid review authors to describe their methods explicitly, and the limitations that time may place on their review [2].

### Objectives

The objectives of this paper are (i) to describe decisions made in the context of a specific review undertaken during unprecedented circumstances, and (ii) to contribute to the discourse surrounding rapid QES.

#### The team and the work process

From inception to publication, our rapid review took 25 days, with the bulk of the work taking place over 2 weeks. Our published review includes a short reflexive account offering insight into the professional backgrounds of the authors and the perspectives we brought to the review process [3]. For our review, four review authors (the ‘core team’) simultaneously conducted all the review processes as opposed to a segmented or parallel approach (i.e. where review authors are allocated different stages of the QES). We return to this point about ‘our process’ throughout this paper and propose that the benefits of this warrant further exploration. Communication between the core team was constant (frequent emails, multiple daily videoconference meetings and text messages). The other members of the team (and the Cochrane community) answered email queries promptly, returned feedback within hours, and were on call to consult on methodological or topic-specific questions. This effort is important to remember but is described rarely in published reviews, and most rapid reviews offer scant testimony to the commitment and hours required of everyone involved.

## Methods

### Formulating a research question

Our research objective was pre-defined and prioritised by Cochrane following a request from the World Health Organisation. This objective encompassed all types of healthcare workers, IPC guidelines and respiratory infectious diseases [3]. These concepts are broad in their meaning and added complexity to the search and the review itself. We used the SPICE (**S**etting, **P**opulation, Phenomenon of **I**nterest, **C**omparison, **E**valuation) [14] criteria to clarify these concepts. Involving a topic expert (XHC) on the team was crucial in efficiently defining our inclusion and exclusion criteria to enable us to conduct a review that was responsive to the COVID-19 pandemic, yet also relevant to other respiratory infectious diseases requiring IPC measures.

### Search

Interim rapid review guidance from Cochrane recommends that an information specialist should be involved [15]. In our review, the information specialist was integral to the team [3], developing a search strategy that was then peer-reviewed by the team, including our methodological expert (AB), who drew on his combined information specialist/systematic review background to optimise the specificity of the search. It was subsequently formally peer-reviewed by a Cochrane information specialist.

The aforementioned scoping review [2] reports considerable variation across the number of electronic databases searched for in rapid QES (ranging from 3 to 7 databases). Due to our restricted timeframe, and guided by Cochrane’s interim rapid review guidance [15], we targeted a single MEDLINE database, informed by estimates of high yields of included studies [16]. We did not include grey literature and record this as a limitation of our work [3]. Whilst we acknowledge that an exhaustive search may not be necessary for QES, we undertook an additional scoping exercise of more than 1500 title and abstracts. We searched the reference lists of key papers [16, 17]. This supplementary searching was helpful as it yielded 30 additional studies for screening [3].

### Selection of studies

Cochrane’s interim rapid review guidance suggests a title and abstract screening approach that involves two screeners in double independent screening for at least 20% of abstracts with conflict resolution and one screener thereafter (using a second reviewer to screen all excluded abstracts to validate the process) [15]. A similar approach is suggested for full-text screening. Most reviews included in the scoping review of rapid QES used single reviewer screening [2]. In our review, the core team double-screened the title and abstracts independently within Covidence (Covidence Systematic Review Software. Available at www.covidence.org). A similar process was applied to full-text screening. Disagreements were resolved by discussion or by involving a third reviewer [3].

On reflection, this approach had two specific benefits. First, constant communication between the core team on how we understood our eligibility criteria helped us to minimise bias at the screening stage. Second, the review authors sensitised themselves to the studies and the data from the screening stage. Potentially, this early immersion facilitated our rapid transition across the QES methods of screening, data extraction and synthesis.

On completion of full-text screening, we identified 36 studies that met our inclusion criteria [3]. Whilst it is not appropriate to prescribe how many studies should be included in a QES [17], we were aware that this number could make it challenging to become sufficiently familiar with the data [18] and so could impair the quality of our analysis. We decided to purposively sample 20 of the studies for inclusion in the synthesis (based on relevance, geographical spread, and depth of insight [19]) [3]. Sampling strategies are increasingly accepted within QES as they seek variation in concepts rather than an exhaustive sample [19]. However, sampling strategies can have important impacts on review findings and benefit from lengthy consideration. The timeframe we had to make this decision was, therefore, not ideal. We have described the sampling strategies we used as a point of reflection, not as prescriptive guidance.

### Data extraction

Cochrane’s interim rapid review guidance recommends that one reviewer extracts the data using a piloted form whilst a second reviewer checks the extracted data for accuracy and completeness [15]. The scoping review of 15 rapid QES noted single reviewer data extraction as the most commonly reported method [2]. For our review, the core members used a data extraction form (Google Forms) designed specifically for this synthesis. The form was piloted on three studies, one reviewer extracted data, which was reviewed for accuracy and completeness by a second member of the team [3]. Following minor revisions to the form, each reviewer extracted data from one study, which was reviewed resulting in final alterations before full data extraction from all included studies. Continuous discussion and moderation across studies were conducted to ensure consistency.

The data extraction form was developed to support the “best fit” framework approach [20] used to analyse and synthesise the evidence (see the ‘Analysis and Synthesis’ section). Categorising the data against a ‘best fit’ framework during the extraction phase, we suggest, allowed a seamless progression to the ‘best fit’ a priori framework approach to analyse and synthesise findings.

### Assessment of methodological limitations

Cochrane’s interim rapid review guidance suggests that quality assessment of included studies is conducted by a single reviewer, with verification of all judgements by a second reviewer [15]. Most of the rapid QES reported in the scoping review [2] conducted a quality assessment (mainly using the Critical Appraisal Skills Programme Qualitative Checklist Tool (CASP) [21]). We, similarly, used an adaptation of the CASP tool (the adapted tool is documented in the published rapid QES). Each included study was appraised independently by two core team members with disagreements resolved through discussion [3]. On reflection, we could have considered a single reviewer assessment with a process of verification. However, we persisted with double, independent assessments in seeking to balance speed with rigour.

### Analysis and synthesis

Cochrane’s interim rapid review guidance on analysis was not considered relevant to our review, given its focus on effectiveness reviews [15]. The scoping review of rapid QES reports use of the same approaches used by regular QES [2]. We used a ‘best fit’ framework approach [20, 22]. This deductive approach is considered by its creators, and others [23], as particularly suitable for rapid synthesis [22], and this guided our decision. This pragmatic method builds on an a priori framework, enriching existing theory, rather than employing a grounded or inductive form of synthesis [24, 25]. We identified a pre-existing framework [26] that we used to analyse the studies under the domains of organisational, environmental and individual factors that can impact IPC guideline adherence.

In keeping with ‘our process’, the core team conducted analysis and synthesis simultaneously. Using the ‘best fit’ framework approach to inform data extraction meant that individual reviewers could analyse the data to populate different domains of the framework. We engaged in several online discussions to ensure consistency across analysis and synthesis, reduce overlap in the findings by mapping the range and nature of reviewed concepts and identify how the themes addressed the review question and review aim [3]. We believe that this contemporaneous critical peer review of synthesised findings made our process more trustworthy, contributing to the coherence and relevance of the findings.

Engaging in all stages of the QES, from screening to synthesis, helped the review authors move between the data and the developing themes more effectively. In our experience, this intense part of the review, given the immersion needed during analysis, requires uninterrupted focus facilitated by conversations conducted in real-time.

### Assessing the confidence in the findings

Cochrane’s interim guidance about assessing the certainty of evidence in rapid reviews suggests a single reviewer approach, with verification from a second reviewer [15]. This topic is not documented in the scoping review of rapid QES [2]. For our review, the core team independently used the Confidence in the Evidence from Reviews of Qualitative Research (GRADE-CERQual) approach to assess confidence in the findings [27]. The review authors initially applied GRADE-CERQual to those findings that were within the same domains they had explored previously in-depth during the synthesis stage. The final assessment was based on a consensus across the core team [3]. Whilst our assessment was rigorous, we did not have time to edit and include full evidence profiles in the published review (the evidence profiles are now complete and are documented in Appendix 2 of the published rapid QES).

Having team members who were familiar with GRADE-CERQual was important for expediting this stage. We were able to refine our approach in line with previous experiences and used a piloting process where each of the core team performed the assessment on three key findings and presented back to the others providing the rationale for the level of confidence we awarded. This pilot exercise was valuable in identifying any ambiguity in our GRADE-CERQual judgements early in the process. The review authors undertaking the GRADE-CERQual assessment had conducted the data extraction and the assessment of methodological limitations in relation to these studies informing the findings. Again, this knowledge and familiarity helped to expedite the process.

### Developing implications for practice

Developing implications for practice was not a specific requirement in the Cochrane interim rapid review guidance. However, we felt that to support decision-makers, this section was critical. Our review team included an editor from the Cochrane Effective Practice and Organisation of Care Group (EPOC) with experience of communicating findings in this way [28], who could prepare this section quickly. This work involved mapping individual review findings into questions for consideration by decision-makers. We also included these questions in an evidence summary for end users [29].

To ensure that this summary was accessible and relatable to end users, we invited 18 nurses, doctors and care home staff from different continents and areas of healthcare to review the summary. Fifteen volunteers responded within 24 h, and minor edits were made accordingly. We sourced volunteers to translate the summary into Spanish, Portuguese, French and Norwegian. Volunteers also translated the plain language summary of the review into five languages. In addition, we recorded a podcast, since translated into multiple languages, developed an infographic and featured in Evidence Aid [30].

These efforts and generosity from others increased the accessibility and usefulness of the rapid QES findings. Future rapid review guidance needs to consider knowledge translation as a key element.

## Discussion

Our first objective in this paper was to describe how we undertook a rapid QES at a unique moment in time. We describe how the process enabled us to meet the remit and timeline of Cochrane’s COVID-19 rapid reviews. Our second objective was to contribute to the discourse surrounding rapid QES. The need for more guidance on rapid QES remains, but such guidance needs to be based on worked examples. This paper may provide a useful framework for others to use and further develop.

We are mindful of the potential criticism that rapid reviewers may privilege processes that are generic across all review types, rather than preserve the philosophical underpinnings of qualitative research [2]. Concerns about superficial QES approaches already exist [31], and this critique must inform the decisions and claims that reviewers make about their outputs. Campbell et al. suggest that concerns about potential limitations must be weighed against the argument that, during periods of limited resources and time, ‘something is better than nothing’ [2]. The limitations of our work are documented here and within the published review [3]. Producing a timely review for the research and healthcare community required some decisions to compromise. We do not suggest that these decisions are easy to make; our team would have enjoyed making methodological decisions under less pressured conditions. However, the limited available time certainly did not restrict the depth of our engagement and discussion. Responding effectively to a question about COVID-19 during a global pandemic necessitates, or at least permits dynamic, pragmatic, and responsive attitudes to guide decision-making. The challenge lies in finding the balance between the time needed for thoughtfulness and the need to respond rapidly.

Our final points remain as reflexive thoughts as we highlight the factors that enabled the completion of our rapid review. The entire process, from initiation to publication took 25 days. Several factors influenced our ability to complete the review within this timeframe. We highlight the broader team of supporters, including the editors, peer reviewers, translators, healthcare workers, end-user stakeholders, and the broader Cochrane community. The membership of our immediate team and the support we received from the wider community was critical in completing and publishing our review within this timeframe. The core team had worked together previously and this also enhanced our completion trajectory. The merits of conducting rapid research with people who recognise each other’s work ethic, skill sets and personalities receive almost no attention in the literature. This should be explored in further methodological discussions. The process we employed relied on the core team moving together through the methods of this QES. We believe this approach called for a frequent and in-depth discussion in real-time. This constant communication (and follow-up consultations with the wider team) also supported the reflexivity required to balance speed with rigour.

International experts in methodology and topic areas joined this team at a time when they were already inundated with commitments to the COVID-19 response. The generosity of everyone to answer this question played a role that cannot be underestimated. In addition, through the Cochrane structure, the team had access to an international network offering specialised methodological and practical support. Cochrane EPOC’s editorial team maintains high levels of QES expertise providing essential guidance for sections of the review, as well as experience in dissemination and end-user input. The Cochrane community was quickly able to identify peer reviewers and copy editors, who contributed within the timeline. We cannot speculate whether such commitment would have been possible at another time, nor the extent to which we could have harnessed these energies outside the exceptional circumstances and urgency of this review question. We are confident that there are lessons here for the future of how evidence syntheses are conducted.

## Conclusion

This paper offers insights into conducting a rapid QES during the COVID-19 pandemic. We have highlighted our decisions, the process we undertook and areas for consideration by the review community. Members of the Oxford Centre for Evidence-Based Medicine have suggested previously that rapid reviews would be more appropriately rebranded as ‘restricted systematic reviews’ [32]. Regardless of the label, we return to an earlier reflection that calendar dates may denote rapid completion, but they do not attest to the extensive hours and intensive effort required; a point of which those who agree to take on such endeavours must be aware!
